# Complex Interactions between GSK3 and aPKC in Drosophila Embryonic Epithelial Morphogenesis

**DOI:** 10.1371/journal.pone.0018616

**Published:** 2011-04-05

**Authors:** Nicole A. Kaplan, Pamela F. Colosimo, Xiaoping Liu, Nicholas S. Tolwinski

**Affiliations:** Program in Developmental Biology, Sloan-Kettering Institute, Memorial Sloan-Kettering Cancer Center, New York, New York, United States of America; University of Colorado, Boulder, United States of America

## Abstract

Generally, epithelial cells must organize in three dimensions to form functional tissue sheets. Here we investigate one such sheet, the Drosophila embryonic epidermis, and the morphogenetic processes organizing cells within it. We report that epidermal morphogenesis requires the proper distribution of the apical polarity determinant aPKC. Specifically, we find roles for the kinases GSK3 and aPKC in cellular alignment, asymmetric protein distribution, and adhesion during the development of this polarized tissue. Finally, we propose a model explaining how regulation of aPKC protein levels can reorganize both adhesion and the cytoskeleton.

## Introduction

Epithelial structures are generated when groups of cells respond to signals defining their fate and organization. One example occurs during the late stages of Drosophila epidermal development. Here signaling prods groups of cells to undergo dramatic morphological changes to form elongated, rectangular cells that secrete actin-based hairs called denticles. Other groups, following different signals, form irregular cells that do not generate denticles [1], [2], [3]. These cells, therefore, translate extracellular signals into morphogenetic changes allowing a close examination of how signaling may influence polarity [4], [5].

Generally, cells in epithelia tend to pack together in roughly hexagonal structures [6]. In contrast to this simple array, morphogenesis in the late epidermis leads to a subset of cells taking on a rectangular, organized morphology. This organization, within the plane of the tissue, depends on the asymmetric distribution of adherens junctions, apical-basal polarity determinants and cytoskeletal components [4], [5], [7], [8], [9], [10], [11]. This tissue is patterned by a variety of signaling pathways including Wnt signaling [12], [13]. The involvement, however, of apical-basal polarity proteins suggests a non-canonical Wnt signal, especially since aPKC has been shown to function in non-canonical Wnt signaling [5], [14], [15], [16], [17].

We investigated the function of aPKC in planar organization of the Drosophila embryonic epidermis. aPKC was enriched at the dorsal/ventral margins of epidermal cells. This distribution was regulated by the Wnt pathway component Glycogen Synthase Kinase 3 (GSK3 or Zw3). Through a genetic approach, we propose a role for GSK3 in linking signaling, to polarity and adhesion.

## Results and Discussion

### The apical polarity protein aPKC is asymmetrically distributed in epidermal cells

**Figure 1:**
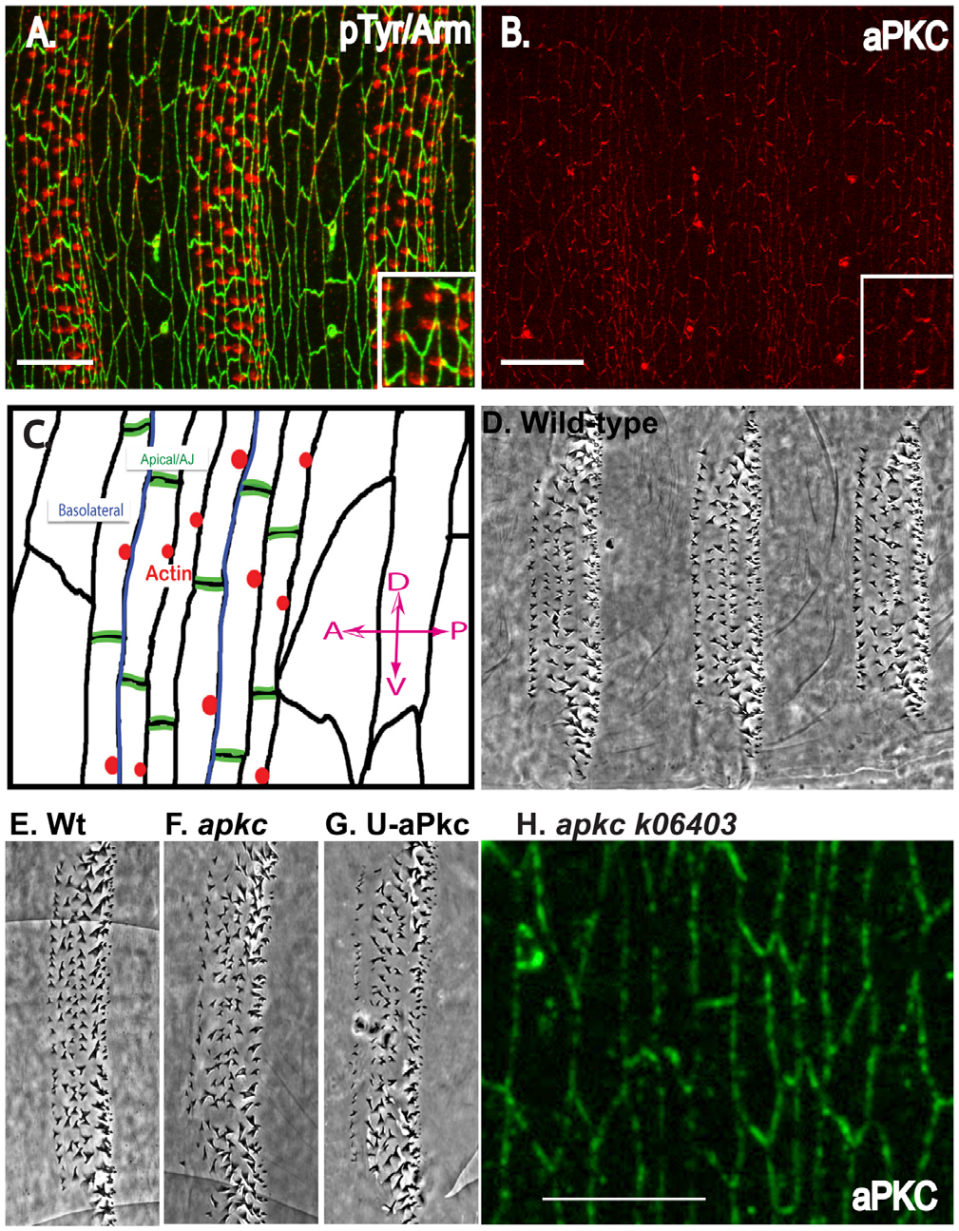
Apical-basal components display asymmetric distribution. (A) Stage 15 epithelium with junctions in green (Arm) and denticle precursors in red (pTyr). Note the rectangular cell shapes of denticle-secreting cells, versus the squamous, irregular shape of cells that do not secrete denticles. Notice also the asymmetric Arm distribution, and actin-based denticle precursors localized to the posterior of cells. (B) A stage 15 ventral epithelium showing polarity protein aPKC enriched at the D/V margins of cells. (C) Schema of a stage 15 embryonic ventral epithelium. The apical components and adherens junction components are asymmetrically distributed to the D/V margins of cells. The baso-lateral components are asymmetrically distributed to the A/P margins of cells. Rectangular cells produce an actin-based precursor at the posterior edge of cells. (D-E) Cuticle of wild-type denticles, with denticle rows properly aligned. (F) Denticle belt of an *aPKC* mutant, which has very slightly misaligned denticle rows. (G) An embryo overexpressing aPKC also shows little to no phenotype, with very mild denticle misalignment. (H) Ventral epithelium of an *aPKC* mutant showing that the aPKC protein is still present at cell membranes in the mutant embryo. Anterior to posterior (A/P) and dorsal to ventral (D/V) directions are shown in the schematic (C) and this orientation is maintained in all figures. Scale bar = 20 µm.

During the final stages of embryonic development, epidermal cells undergo a morphogenetic change that transforms a disorganized epithelium into a structured, aligned, and organized epidermis. The rectangular cells go on to secrete actin-based denticles in a regular pattern (Fig. 1A, D). The great surprise, however, was the finding that this process requires the asymmetric distribution of apical-basal determinants and adherens junctions within the plane of the epithelium while maintaining perfect apical-basal polarity [4], [5], [9], [10], [11]. As the process begins, polarity determinants are asymmetrically distributed within the rectangular cells; the baso-lateral components are enriched on the anterior/posterior (A/P) cell margins directly opposite to apical polarity determinants, which are enriched on the dorsal/ventral (D/V) cell margins (Fig. 1 B and C). Adherens junctions co-localize with apical determinants at D/V cell margins (Fig. 1C) [5], [7], [8], [10].

We focused on the apical kinase aPKC because it is a key regulator of polarity and is enriched at D/V margins (Fig. 1B)[18]. We investigated its role in morphogenesis by looking at *aPKC* mutant embryos, however we did not observe a strong phenotype in denticle formation or alignment. In *aPKC* mutants there was only a mild effect on denticles (null or amorphic *aPKC* mutant, Fig. 1F), and similarly overexpression of aPKC showed little effect (Fig. 1G compare to Fig. 1E) [19], [20]. This is likely due to an incomplete loss of aPKC protein in mutant embryos due to maternal RNA loading (Fig. 1H shows aPKC protein present in *aPKC* null zygotic mutants even at late stages); however, we were unable to analyze maternal mutants at late stages as they disintegrate during early gastrulation processes [18]. In order to overcome the weakness of the phenotype, we turned to *GSK3* mutants as loss of this kinase can enhance aPKC levels and activity [8].

### GSK3 regulates the asymmetric distribution of aPKC

**Figure 2:**
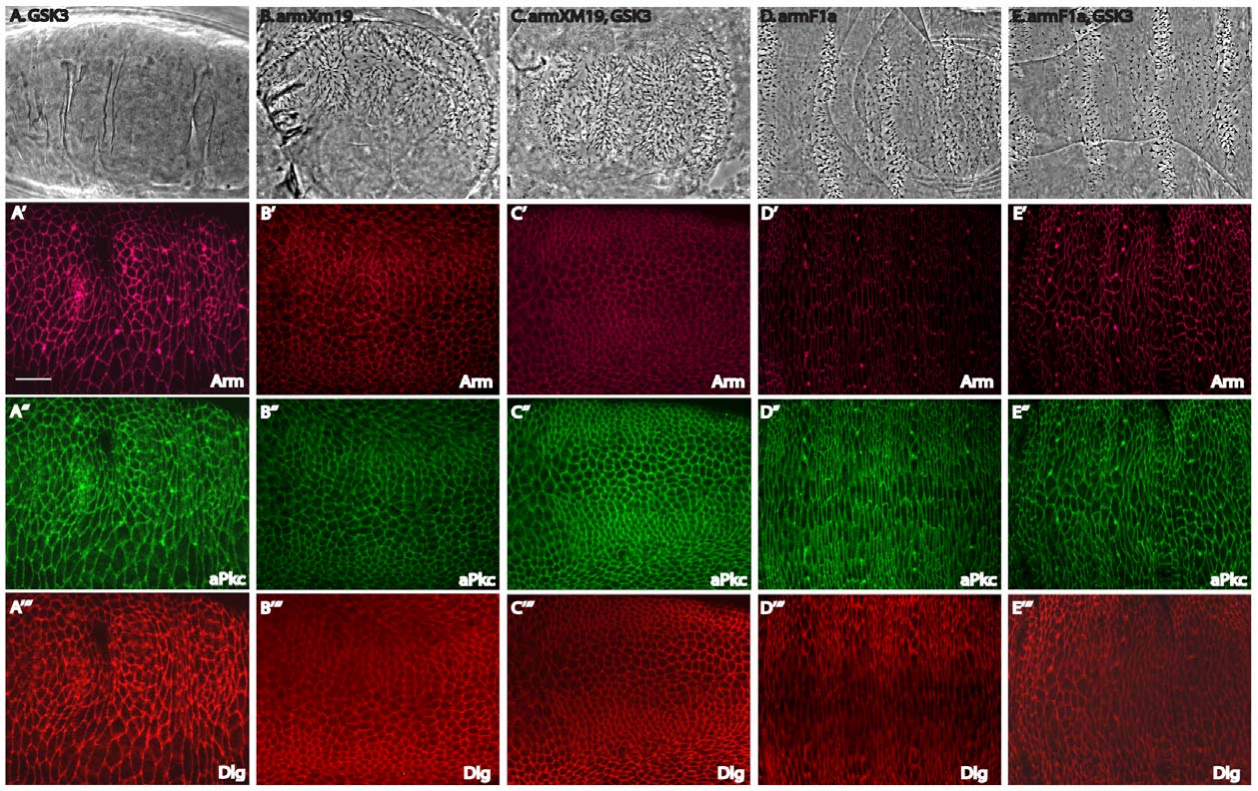
Patterning is required for asymmetric distribution of adherens junctions and polarity components. All mutants represent germline clone embryos, or maternal and zygotic (M/Z) mutants unless otherwise noted. (A) Mutations in *GSK3* (the *Drosophila* allele is named *zw3^M11-1^*) led to a compete loss of patterning due to hyperactivation of the canonical Wnt pathway, and a completely naked cuticle. (B) *arm^XM19^* mutants led to the opposite phenotype in which the cuticle was completely covered with denticles as canonical Wnt signaling is completely lost. (C) Additional mutation of *GSK3* along with *arm^XM19^* showed an identical phenotype to *arm^XM19^* alone. (D) The weaker *arm^F1a^* mutant blocked canonical Wnt signaling to a certain extent, but some patterning remained. (E) The additional mutation of *GSK3* along with *arm^F1a^* showed a phenotype similar to *arm^F1a^* alone, with cell shapes and alignment appearing slightly more irregular. (A'–E') Staining for the junctional component Arm in the various mutants. Though levels are reduced in the *arm* mutants, sufficient protein remains to sustain adherens junctions and tissue integrity. Notice that the staining is symmetric around cells in (A') through (C') but appears somewhat more polarized to D/V margins in (D') and (E'). (A''–E'') Staining for the apical marker aPKC showing that it is generally uniform except for a mild enrichment in *arm^F1a^* alone (D''). (A'''–E''') Staining for the basolateral marker Dlg again showing that it is not polarized in the mutants aside from an A/P enrichment in *arm^F1a^* alone (D''). Scale bar = 20 µm.

**Figure 3:**
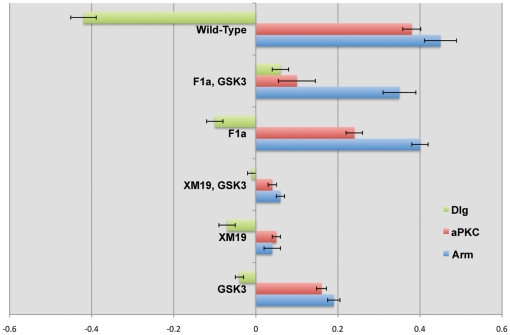
Mutation phenotypes quantified. Graph representing quantified polarization of Arm, aPKC and Dlg in the mutants presented in figure 2. Representation uses a logarithmic scale to allow for positive (D/V polarization) and negative (A/P polarization) values in the representation.

aPKC regulates adhesion, and its protein levels are regulated by GSK3 phosphorylation and ubiquitin-mediated proteasomal degradation** [8]. Based on this, we investigated whether GSK3's regulation of aPKC affects epidermal morphogenesis. Although various targets for GSK3 have been proposed [21], the clearest phenotype in loss-of-function *GSK3* mutant embryos is the ectopic activation of canonical Wnt signaling causing all epithelial cells to switch to the naked cell fate [22]. These embryos lack pattern and obvious polarity, their cells do not secrete denticles, and all examined markers appear uniform (null or amorphic *GSK3* maternal and zygotic mutant, Fig. 2 A-A'''). In order to assess the effects of *GSK3* deletion in denticle-producing cells, we restored denticles by attenuating canonical Wnt signaling with a downstream mutation in *armadillo* (*arm*). Our genetic system utilized two *arm* mutations: *arm^F1a^* blocks most Wnt function, whereas *arm^XM19^* blocks Wnt signals completely [23], [24]. In the stronger *arm^XM19^* mutant, patterning was abolished, all cells had a similar shape, secreted a denticle, and the planar distribution of all markers was uniform around cell margins (Fig. 2 B-B'''). In the weaker *arm^F1a^* mutant, some patterning was maintained, and cell shape changes were only mildly disrupted (Fig. 2 D-D'''). Cells were rectangular and asymmetry of markers was moderately maintained. Into these mutants we introduced *GSK3* mutations, and in neither case *arm^XM19^*, *GSK3* (Fig. 2 C-C'''), nor *arm^F1a^*, *GSK3* (Fig. 2 E-E''') did the additional mutation have an effect on epithelial patterning. To clarify whether markers were asymmetrically distributed, we quantified the fluorescent signals of Arm, aPKC and Dlg in mutant embryos and compared it to wild-type embryos (Fig. 3). These results suggested that although patterning is similar, cell shapes and asymmetry of markers appeared more disrupted in the *arm^F1a^, GSK3* double mutants when compared to *arm^F1a^* alone.

### Distinct roles of aPKC in apical-basal polarity and morphogenesis

**Figure 4:**
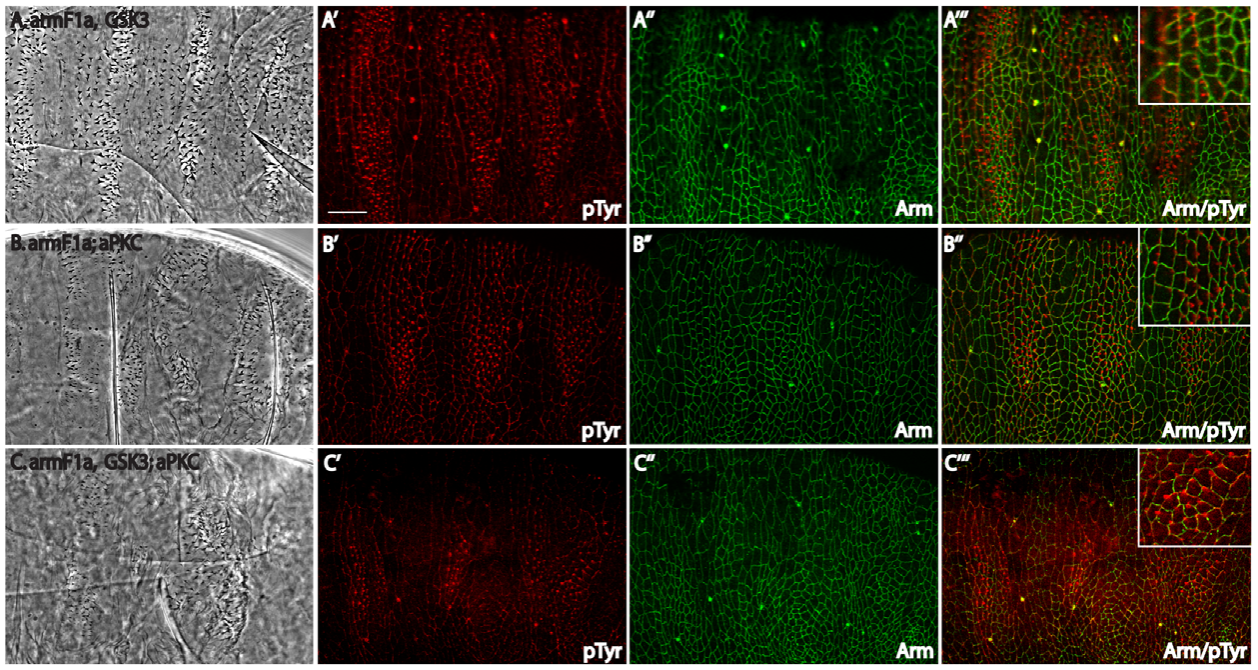
Epithelial organization is disrupted by loss of aPKC. (A) Double mutant for *arm^F1a^* and *zw3^M11-1^* (M/Z) retained some patterning and denticles lined up in rows. (B) *arm^F1a^* (M/Z) and *aPKC^k06403^* (Z) double mutant has disrupted row alignment, but some patterning is maintained. The phenotype was observed in 8/78 embryos with 12.5% predicted for full penetrance. (C) In the *arm^F1a^*, *zw3^M11-1^* (M/Z); *aPKC* (Z) triple mutant, denticle arrangement is severely disrupted and rows are not apparent. The phenotype was observed in 10/98 embryos with 12.5% predicted for full penetrance. (A'-A''') *arm^F1a^* and *zw3^M11-1^* (M/Z) double mutant stained for pTyr in red and Arm in green. Though the cell shapes are affected, the general organization of cell rows remains. (B'-B''') *arm^F1a^* (M/Z) and *aPKC^k06403^* (Z) double mutant stained for pTyr in red and Arm in green. Again, the cell shapes are mildly affected but cell alignment is maintained. (C-C''') *arm^F1a^*, *zw3^M11-1^* (M/Z); *aPKC* (Z) triple mutant stained for pTyr in red and Arm in green. These embryos showed a stronger disruption of cell shapes and cell alignment, and denticle precursors appeared in swirls instead of rows—see especially insets. Scale bar = 20 µm.

Although the *arm* mutant phenotypes were not considerably altered by loss of *GSK3*, the mild phenotype of the *arm^F1a^*, *GSK3* double mutant provided a sensitized background in which we could further assess the role of aPKC in morphogenesis. We introduced *aPKC* null, zygotic mutations into both *arm^F1a^*, *GSK3* (hypomorphic *arm* and null *GSK3* both maternal and zygotic mutants) and *arm^F1a^* mutants. *arm^F1a^*; *aPKC* double mutants maintained some patterning, and while cell shapes were somewhat disrupted, cells appeared to align into rows (Fig. 4A-B). However, in the *arm^F1a^*, *GSK3; aPKC* triple mutant, denticle and cell alignment were significantly disrupted (Fig. 4C). Specifically, proper cell shapes and cell alignment were lost, and instead of organization into even rows, cells curved and formed denticle swirls. Together, our results point to GSK3 and aPKC regulating cellular alignment in the epithelium.

**Figure 5:**
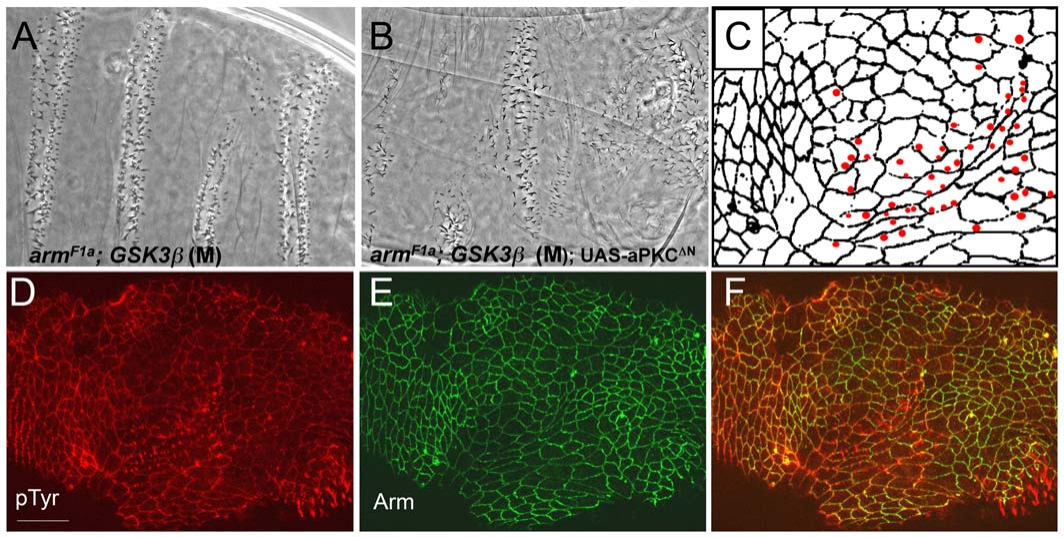
Increased levels of aPKC cause morphogenesis defects. (A) *arm^F1a^*, *zw3* maternal only mutants (M) display mild patterning defects, but denticles are still present in proper rows. (B) *arm^F1a^*, *zw3* maternal mutants that express the daGAL4>UAS-aPKC^ΔN^ transgene display severe defects, as denticle organization is completely lost. (C) Schematic of *arm^F1a^*, *zw3* maternal mutants expressing the daGAL4>UAS-aPKC^ΔN^ transgene showing the severe cell shape and alignment defects and random denticle placement (red dots) seen in these embryos. (D–F) Staining of the *arm^F1a^*, *zw3*maternal mutants expressing aPKC^ΔN^ with pTyr in red and Arm in green. (D) pTyr shows the random placement of denticle precursors, and the denticle swirls rather than proper rows. (E) Arm localization shows the severe cell shape and alignment defects. (F) Merged view of pTyr and Arm localization shows that denticle placement is random within cells and the dorsal/ventral margins of denticle-producing cells do not display the proper enrichment of Arm protein. Scale bar = 20 µm.

aPKC expression in *GSK3* mutants leads to embryos with severe apical-basal defects [8]. It was therefore not possible to assess denticle organization in these embryos. Denticle development did, however, proceed in embryos only missing the maternal dose of *arm^F1a^* and *GSK3* Fig. 5A). In this mutant, we expressed *aPKC^ΔN^*, a gain-of-function form of aPKC where the N-terminal domain normally used for binding to Par-6 and restricting the localization and activity of aPKC is deleted [25]. These embryos displayed randomly oriented denticles (Fig. 5B, phenotype was observed in 16/115 (25% Expected)), instead of the relatively uniform pattern seen in embryos without the activated aPKC^ΔN^ transgene (Fig. 5A). We examined these defects further by looking at the localization of phospho-Tyrosine (pTyr), a convenient marker of denticle precursors, and Arm to highlight cell-cell junctions (Fig. 5D–F, pTyr is present in all cells, but forms puncta in denticle producing cells). Again, similar to the mutants discussed above, we found that cells secreting denticles were not rectangular or aligned in regular rows (Fig. 5F, schematic in Fig. 5C). This effect was specific to *GSK3 *mutants as neither expression of *aPKC^ΔN^* in otherwise wild-type embryos, nor in *arm^F1a^* mutant embryos showed an effect on denticle organization (Fig. 1G and not shown). Therefore, by differentially altering the levels of aPKC we find that aPKC performs multiple functions in cell polarity. One caveat is that since in *aPKC^ΔN^* the Par-6 binding region is deleted, it is not restricted to the apical compartment through Par-6 binding, therefore the effect we observe may occur in another cellular compartment.

### aPKC refractive to GSK3 phosphorylation has morphogenesis defects

**Figure 6:**
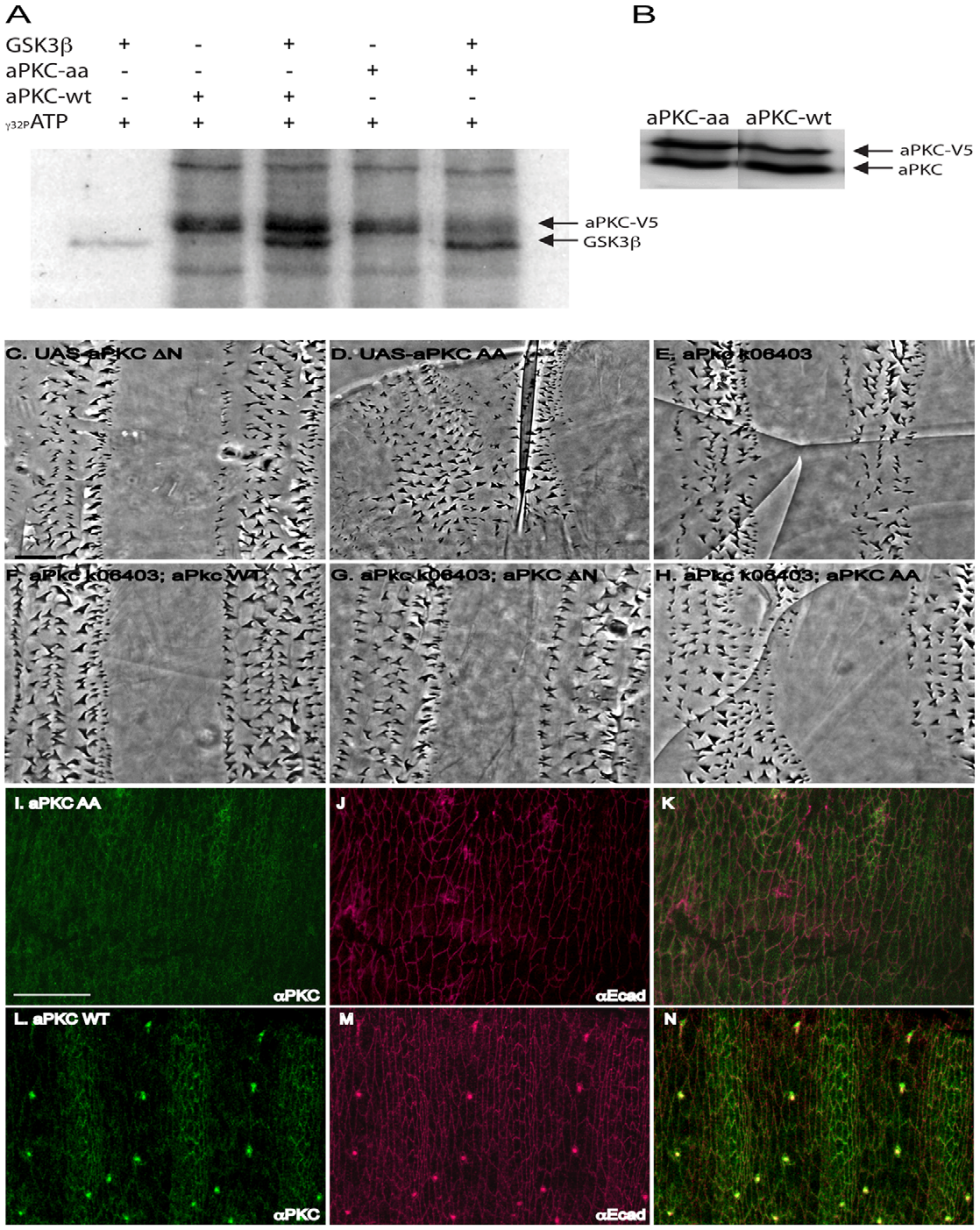
An unphosphorylatable form of aPKC causes polarity defects. (A) Anti-V5 antibody was used to immunoprecipitate V5-aPKC^WT^ and V5-aPKC^AA^ from HeLa cell extracts. Kinase assays using these proteins show some level of autophosphorylation, but V5-aPKC^AA^ is phosphorylated by GSK3 at much lower levels compared to aPKC^WT^. (B) Western blot shows comparable levels of V5-aPKC^WT^ and V5-aPKC^AA^ expression in HeLa cell extracts. (C) Embryos expressing the daGAL4>aPKC^DN^ transgene do not display severe denticle placement defects. (D) In embryos expressing the daGAL4> aPKC^AA^ transgene, denticles are randomly polarized. (E)Cuticles of *aPKC^k06403^* zygotic mutant embryos are normally patterned, and display few denticle arrangement defects. (F) Expression of daGAL4>aPKC wild-type or (G) daGAL4>aPKC^DN^ in *aPKC^k06403^* mutant embryos does not affect cuticle patterning. (H) Expression of daGAL4>aPKC^AA^ in an *aPKC^k06403^* mutants results in denticle polarization defects. Expression of daGAL4>aPKC^AA^ in wild-type embryos (I–K) results in uniform aPKC expression (I). (J) E-cadherin expression shows cell shapes. (K) Merged view. Expression of daGAL4>aPKC^WT^ (L–N), does not affect the striped expression of aPKC (L) stripes. (M) E-cadherin expression shows that cell shapes are not affected. (N) Merged view. Scale bar = 20 µm.

To further investigate the interaction between aPKC and GSK3, we searched aPKC for GSK3 consensus phosphorylation sites and found two putative target residues. We constructed a transgene of *aPKC* (*aPKC^AA^)* carrying point mutations at two predicted GSK3 consensus sites (S330A and T422A). In order to test if these residues are phosphorylated by GSK3, we expressed Drosophila *aPKC* and *aPKC^AA^* in HeLa cells. Following immunoprecipitation with a V5 affinity tag antibody, we conducted kinase assays with the two forms of the protein and recombinant GSK3. Though there is a low level of autophosphorylation with both proteins (as seen in lanes 2 and 4 where there is no GSK3 added), the wild-type version of aPKC is phosphorylated by GSK3, whereas aPKC^AA^ is not (compare lane 3 to lane 5, Fig. 6A). Western blotting showed that comparable amounts of aPKC^AA^ and aPKC^WT^ protein were present in the HeLa cell extracts (Fig. 6B).

Since the aPKC^AA^ protein is refractive to GSK3 phosphorylation, we hypothesized that expression of aPKC^AA^ should be similar to that of endogenous aPKC protein in the absence of GSK3 kinase activity. Wild-type aPKC appears in a striped pattern due to GSK3-mediated degradation [8], but expressed aPKC^AA^ protein does not form stripes (Fig. 6I). To test if this expression pattern is an artifact of overexpression, we also expressed wild-type aPKC under identical conditions, and observed striped expression of aPKC (Fig. 6L). Co-staining with E-cadherin reveals aPKC upregulation in the rectangular cells (Fig. 6M-N). The lack of striped aPKC^AA^ suggests it is refractive to down-regulation by GSK3[8] Taken together, these data point to aPKC being a target of GSK3.

In order to test if these aPKC residues are required for polarity, we expressed *aPKC^AA^* in wild-type embryos. *aPKC^AA^* did not cause any apparent apical-basal phenotype, but in contrast to *aPKC^ΔN^* (Fig. 6C), *aPKC^AA^* did cause denticle alignment defects (Fig. 6D). We next expressed *aPKC^AA^* in embryos lacking *aPKC* function. Drosophila embryos with a loss-of-function allele of *aPKC* develop normally through early stages of embryogenesis, though many die before hatching [19], [20]. As shown in Fig. 6E, denticle organization defects due to loss of *aPKC* are mild, and are rescued by expression of both wild-type *aPKC* (Fig. 6F) and *aPKC^DΔ^* (Fig. 6G). However, expression of *aPKC^AA^* leads to some denticle orientation defects (Fig. 6H), although the severity is lower than that observed for *GSK3* mutants (Fig. 5B). This is likely due to the mismatched nature of our genetic backgrounds with maternal and zygotic mutants used, but taken together the results suggest a role for aPKC phosphorylation in epithelial morphogenesis.

### A genetic model

Previous studies have suggested roles for apical-basal polarity components in several planar polarity processes and stem cell divisions [26], [27], [28], [29], [30]. Our findings define roles for polarity determinants in planar polarity and epidermal cell morphogenesis. Apical polarity proteins like aPKC are polarized and establish domains on the D/V cell margins leading to an upregulation of adherens junctions, and exclusion of the basal-lateral determinants [4], [5]. This is consistent with the current model of apical-basal polarity, which posits that apical components lead to the localization of junctions and compete with the basal-lateral components to establish independent domains within a cell [31], [32], [33]. We show that such domains are established within the plane of the epithelium across many cells, thus raising the possibility that this co-opted apical-basal polarity mechanism regulates morphogenesis in this tissue. For the exclusion model to work properly the levels of apical and basal determinants must be tightly regulated. Therefore, GSK3 plays a crucial role in maintaining the levels of aPKC, although the symmetry-breaking event remains unclear. Future experiments will have to explain how polarity is established; nevertheless, the interaction between polarity, adhesion and the Wnt pathway may have implications for how cancer cells escape tissues during metastasis or are maintained asymmetrically as stem cells [34].

The major caveat of these experiments pertains to our attempt to address these issues *in vivo*. To accomplish this, we must use complex genetic approaches. This is not a problem for genes with only one function, however here we have concentrated on GSK3a gene with myriad functions. For example, one recent study identified 42 direct phosphorylation targets [21]. In order to study GSK3's interaction with aPKC, we blocked GSK3's best-studied function in canonical Wnt signaling, but this leaves many others. Despite this caveat, our findings are most simply explained through a role for GSK3in regulating epithelial morphogenesis through its interaction with aPKC. We cannot, however, exclude other explanations as we have not examined the roles of other GSK3 targets.

## Materials and Methods

### Crosses and expression of UAS constructs

Maternally mutant eggs were generated by the dominant female sterile technique [35]. Oregon R was used as the wild-type strain. Please see Flybase for details on mutants used (flybase.bio.indiana.edu). *aPKC^k06403^* is a P-element insertion that behaves like a null mutation [19], [20]. Other mutants used were the amorphic *zw3^M11-1^*, the partial loss-of-function *arm^F1a^* (mutation leads to Arginine 394 being mutated to a Histidine) that reduces Arm's affinity toward TCF, and the strong hypomorph *arm^XM19^* that contains a stop codon at the end of repeat twelve (stop codon introduced after amino acid 680), deleting the entire C-terminal region preventing Wg signaling [24]. For expression experiments, the armadillo and daughterless-GAL4 drivers were used.

New aPKC constructs: we mutated two consensus GSK3 (Ser330 and Thr422 according to the Drosophila aPKC coding sequence) sites in aPKC to alanines using double strand mutagenesis (QuickChange, Stratagene), and cloned this otherwise full-length aPKC construct into pUASt vector using Gateway technology (Invitrogen). The UAS-aPKC^ΔN^ consisting of amino acids 180-606 [25] was used for genetic crosses.

All X-chromosome mutants use FRT 101. The following crosses were conducted:

1. *zw3^M11-1^* FRT101/ovoD1 FRT101
2. *arm^XM19^* FRT101/ovoD1 FRT101
3. *arm^XM19^ zw3^M11-1^* FRT101/ovoD1 FRT101
4. *arm^F1a^* FRT101/ovoD1 FRT101
5. *arm^F1a^ zw3^M11-1^* FRT101/ovoD1 FRT101
6. *arm^F1a^ zw3^M11-1^* FRT101/ovoD1 FRT101
7. *arm^F1a^* FRT101/ovoD1 FRT101; *aPKC^k06403^*/+ **X** *aPKC^k06403^*/+ males
8. *arm^F1a^ zw3^M11-1^* FRT101/ovoD1 FRT101; *aPKC^k06403^*/+ **X** *aPKC^k06403^*/+ males
9. *arm^F1a^ zw3^M11-1^* FRT101/ovoD1 FRT101; hs-FLP; arm- Gal4 females **X** UAS-aPKC^ΔN^ males
10. *arm^F1a^* FRT101/ovoD1 FRT101; hs-FLP; arm-Gal4 females **X** UAS-aPKC^ΔN^ males
11. *aPKC^k06403^* G13 FRT/OvoD1 FRT G13 **X** *aPKC^k06403^* G13 FRT/CyO males
12. *aPKC^k06403^*/CyO, twi-GFP; daGAL4 **X** *aPKC^k06403^*/Cyo, twi-GFP; UAS-aPKC (WT, ΔN, AA) males for zygotic mutant study in Figure 5.

X-chromosomes were marked with the *yellow* mutation to simplify analysis. Live GFP selection was used for second chromosome homozygous mutant selection. For all crosses more than 100 embryos were analyzed in multiple, separate experiments (n>100).

### Antibodies and Immunofluorescence

Embryos were fixed with Heat-Methanol treatment [36] or with heptane/4% formaldehyde in phosphate buffer (0.1 M NaPO4 pH 7.4) [23], [37], [38]. The antibodies used were: anti-Dlg (mAb 4F3, Developmental Studies Hybridoma Bank (DSHB) developed under the auspices of the NICHD and maintained by The University of Iowa, Department of Biological Sciences, Iowa City, IA 52242), α-Catenin (ratAb Dcat1 DSHB), anti-Armadillo (mAb N2 7A1, DSHB), rabbit anti-Armadillo [39], rabbit and goat anti-aPKCζ, and anti-phospho-tyrosine pY99 (Santa Cruz Biotechnology). Immunofluorescence, detection and image processing as described in [10].

### Western Blotting

Embryos were lysed in extract buffer (50 mM Tris pH 7.5, 150 mM NaCl, 1% NP-40, 1 mM EDTA, 10% Glycerol, Complete Mini Protease, Sigma). The extracts were separated by 7.5% SDS-PAGE, and blotted as described in Peifer et al. (1994). Extracts were normalized using the BCA assay (Novagen). Overnight embryo collections were used to make extracts for Western blots. Kinase assays were performed on both recombinant proteins and proteins immunoprecipitated from embryonic extracts. Recombinant proteins were either purchased from Cell Signaling Technologies (GSK3β and PKCζ) or prepared from bacterial lysates [40]. aPKC phosphorylation was assayed as described in [27].

HeLa cell transfections were performed by standard methods. aPKC full length and aPKC^AA^ were recombined into pDEST40 a vector with 6XHis and V5 tags as C-terminal fusions (Invitrogen). Proteins were immunoprecipitated with the V5 antibody (Invitrogen), and subjected to kinase assays as above.

### Fluorescence Quantification

The intensity of fluorescent staining was measured similar to the procedure used in Harris and Peifer, 2007 [20]. Mean intensity was calculated using Image J software (NIH) for lines that were 1.5–3.0 microns in length at the dorsal-ventral and anterior-posterior edges of cells in denticle-producing rows 2–5. In addition, mean intensity was measured within the cytoplasm of each cell. This background measurement was subtracted from the measurements at the dorsal-ventral and anterior-posterior edges and the ratio between these corrected measurements (dorsal-ventral over anterior-posterior) was calculated. If there was no difference in intensity between edges, we expect a ratio of 1. The ratios were graphed on a logarithmic scale to allow for positive and negative values in the presentation. For measurements 3 to 5 embryos and 10 to 40 cells per embryo were used to obtain intensities.
